# Reactive oxygen species stress increases accumulation of tyrosyl-DNA phsosphodiesterase 1 within mitochondria

**DOI:** 10.1038/s41598-018-22547-8

**Published:** 2018-03-09

**Authors:** Hok Khim Fam, Kunho Choi, Lauren Fougner, Chinten James Lim, Cornelius F. Boerkoel

**Affiliations:** 1 BC Children’s Hospital Research Institute, University of British Columbia, Vancouver, Canada; 2 Department of Medical Genetics, University of British Columbia, Vancouver, Canada; 3 Department of Pediatrics, University of British Columbia, Vancouver, Canada

## Abstract

Tyrosyl-DNA phosphodiesterase 1 (Tdp1) is a nuclear and mitochondrial protein that in nuclei and *in vitro* repairs blocked 3′ DNA termini such as 3′ phosphotyrosine conjugates resulting from stalling of topoisomerase I-DNA intermediates. Its mutation also causes spinocerebellar ataxia with axonal neuropathy type 1 (SCAN1). Because Tdp1 colocalizes with mitochondria following oxidative stress, we hypothesized that Tdp1 repairs mitochondrial DNA (mtDNA) and that mtDNA damage mediates entry of Tdp1 into the mitochondria. To test this, we used *S*. *cerevisiae* mutants, cultured mouse and human cells, and a Tdp1 knockout mouse. H_2_O_2_- and rotenone-induced cellular and intramitochondrial reactive oxygen species (ROS) activated oxidant-responsive kinases P38 and ERK1, and the translocation of Tdp1 from the nucleus to the mitochondria via the TIM/TOM complex. This translocation occurred independently of mtDNA. Within the mitochondria, Tdp1 interacted with Ligase III and reduced mtDNA mutations. Tdp1-deficient tissues had impaired mitochondrial respiration and decreased viability. These observations suggest that Tdp1 maintains mtDNA integrity and support the hypothesis that mitochondrial dysfunction contributes to the pathology of SCAN1.

## Introduction

Tyrosyl-DNA phosphodiesterase 1 (Tdp1) is a 3′-DNA phosphodiesterase that participates in DNA repair. It resolves stalled topoisomerase I-DNA complexes by cleavage of the 3′-phosphotyrosine bond. Less characterized functions of Tdp1 include the processing of endogenous 3′-DNA lesions such as alkylated bases, phosphoamides and chain-terminating nucleosides^1–8^.

Tdp1 colocalizes with mitochondria in human and mouse tissue and in cultured human and mouse cells^9,10^. We previously showed that mitochondrial colocalization of Tdp1 is enhanced by exposure to hydrogen peroxide, suggesting that Tdp1 enters the mitochondria in response to mitochondrial DNA (mtDNA) stress and mediates mtDNA repair^9–11^.

Compared to nuclear DNA, maintenance of mtDNA has several challenges. First, 95% of mtDNA is gene coding, whereas approximately 1–2% of nuclear DNA is^12^. Second, compared to nuclear DNA, mtDNA has increased exposure to mutagens because it lacks protective histones and DNA compaction^13^. Third, mtDNA is in close proximity to the reactive oxygen species (ROS) generated by mitochondrial respiration^14^. Fourth, compared to the nucleus, mitochondria lack certain DNA repair pathways^15^.

Mitochondria produce oxidative agents as a by-product of electron transport chain activity. The generation of volatile oxidants during respiration causes polymerase-blocking oxidative lesions in mtDNA such as thymine glycols and 8-oxoguanine^16,17^. The 8-oxoguanine lesions cause guanine to thymine transversions^18^ and mitochondrial dysfunction by mutation of mtDNA-encoded proteins, tRNAs, and rRNAs. Mitochondrial lysates from breast cancer cells demonstrate base-excision repair (BER) activity^9^, a repair mechanism for oxidative lesions. Because Tdp1 participates in nuclear BER and colocalizes with mitochondria, it has been hypothesized that Tdp1 also functions in mitochondrial BER^6,9,10^.

H_2_O_2_-induced ROS activates stress-activated protein kinase (SAPK) signaling for import of proteins into the mitochondria. The activation of P38 MAP kinase mediates translocation of Bax to mitochondria where it oligomerizes with Bak and forms a pore on the mitochondrial outer membrane, allowing the release of cytochrome C and other pro-apoptotic proteins^19^. H_2_O_2_ also mediates crosstalk between mitochondria and neighboring organelles and, by peroxidation of mitochondrial membrane lipids, regulates secondary redox signaling in the cytoplasm^20,21^.

Herein we show that within *S*. *cerevisiae*, mouse and human cells, ROS enhances Tdp1 entry into the mitochondrial matrix and that this is independent of new protein synthesis and mtDNA. We also find that intramitochondrial ROS is a signal for translocation of Tdp1 out of the nucleus and acts through P38 and ERK1. Within mitochondria, Tdp1 interacts with the BER protein Ligase III. Consistent with a role for Tdp1 in mtDNA repair, embryonic fibroblasts and myoblasts derived from *Tdp1*^−/−^ mice have more mtDNA lesions and impaired mitochondrial respiration compared to those derived from *wt* tissues.

## Materials and Methods

Some methods and materials are defined below, whereas others are described in the Supplementary Methods.

### Animal subjects

All mice used in this study were housed, bred, sacrificed and studied in accordance to approved and ethical guidelines of the Institutional Review Board of University of British Columbia (protocol # A15–0017). *Tdp1*^−/−^ mice used in this study were generated as previously described^22^.

### Cell culture

Human dermal fibroblasts were obtained from DSMZ in Germany. Mouse embryonic fibroblasts were harvested from mouse embryos as previously described^22^. Both cell lines were cultured in DMEM (Gibco BRL Life Technologies) supplemented with 10% heat-inactivated FBS (Hyclone) and 1% antibiotic-antimycotic (Gibco BRL Life Technologies). All cells were grown at 37 °C with 5% CO_2_ in a humidified environment.

### MTT assay

Cell proliferation and viability were measured by MTT assay as previously described^23^. Briefly, 5 × 10^3^ cells were plated in each well of a 96-well plate and cultured in phenol red-free media. Following treatment of cultured cells, the culture media was supplemented with 10% MTT and incubated for 3 hours. This was followed by a 30-minute incubation in DMSO. Spectrophotometry was done at 565 nm using the Wallac VICTOR2 Multilabel Plate Reader (Beckman-Coulter).

### Immunoblotting

Immunoblotting was performed as described^22^. Briefly, cells were lysed in SDS-sample buffer and denatured by boiling for 5 minutes. The lysate was fractionated on a 10% or 15% gel by SDS-PAGE and transferred to a PVDF membrane. The membrane was incubated overnight at 4 °C with blocking solution (0.2% I-Block (Applied Biosystems) and 0.1% Tween 20 in 1x PBS) and gentle agitation. The membrane was then incubated overnight at 4 °C with primary antibodies diluted in blocking buffer. After washing, the membranes were incubated with secondary antibodies for 1 hour at room temperature. The signal was detected with the CDP-Star^TM^ chemiluminescent system (Applied Biosystems). The antibodies used for immunoblotting are listed in Supplementary Table 1.

### Quantitative immunoblotting

Quantitative fluorescent immunoblotting was performed as previously described^24^. Briefly, 20 µg of total protein was separated by SDS-PAGE on a 10% gel and then transferred to a PVDF membrane. Subsequently, the membrane was rocked with a blocking solution (1:1 mixture of LI-COR Odyssey blocking buffer and 1 × phosphate buffered saline) for 1 hour at room temperature and incubated for 1 hour at room temperature with primary antibodies diluted in blocking solution and 0.01% Tween 20 (Supplementary Table 1). After washing four times for 5 minutes in washing solution (1x PBS, 0.01% Tween 20), the membrane was incubated for 1 hour at room temperature with secondary antibodies diluted in blocking solution plus 0.01% Tween 20. After washing four times for 5 minutes in washing solution, a final 5-minute wash was performed with 1 × PBS. A laser intensity of 5 for the appropriate channels was used as default when the membrane was scanned. The IRDye® 800 CW Goat anti-Mouse IgG and IRDye® 800 CW Goat anti-Rabbit IgG antibodies were used at a dilution of 1:10,000.

### Immunofluorescence microscopy

Immunofluorescence microscopy was performed as described^22^. Briefly, approximately 5 × 10^5^ cells were grown on a cover slip. Human or mouse cells were washed with 1 × PBS and fixed with 4% paraformaldehyde (PFA) in phosphate buffer (PB, 22.46 mM NaH_2_PO_4_, 77.54 mM Na_2_HPO_4_, pH 7.4) for 15 minutes. Cells were permeablized with 0.5% Triton X-100 in 1 × PBS for 15 minutes, blocked for 1 hour at room temperature with blocking buffer (20% horse serum, 1% casein in 1 × PBS), and then incubated overnight at 4 °C with the primary antibodies (Supplementary Table 1). All images were acquired using the Leica TCS SP5 II confocal microscope.

### Immunoprecipitation of Tdp1

Briefly, we used the FLAG^®^ HA Tandem Affinity Purification Kit (Sigma-Aldrich) to immunoprecipitate Tdp1-interacting proteins in the mitochondria of cultured MEFs. Preparation of mitochondrial lysate was performed as described in the Supplementary Methods. Preparation and immobilization of the FLAG and HA-tagged Tdp1 protein, as well as capture and elution of prey protein was performed as per the manufacturer’s protocol. The presence of Tdp1-interacting proteins in the eluate was determined by SDS-PAGE and immunoblotting.

### Yeast strains

Yeast strains were cultured as described in their source publications. Single gene mutants were obtained from the yeast deletion collection^25^. The TDP1-GFP strain was obtained from the Yeast GFP collection^26^. Temperature-sensitive knockout strains of essential genes TIM23 and MIA40 were obtained from the temperature sensitive mutant repository^27^.

### Transfection of cultured cells with siRNA

Human dermal fibroblasts were transfected with ERK1 siRNA (Thermo Scientific/Dharmacon; L-003592-00-0005), p38 siRNA (Cell Signaling; 6562 S) or Tim23 siRNA (Thermo Scientific/Dharmacon; L-190121-00-0005). Rho-zero MEFs were transfected with Erk1 siRNA (Thermo Scientific/Dharmacon; L-040126-00-0005) or p38 siRNA (Cell Signaling; 6417 S). All siRNAs were transfected using Lipofectamine 3000 (Thermo Fisher Scientific) according to the manufacturer’s protocol and incubated in appropriate culture media for 48 hours prior to experimentation. Cells were transfected with non-targeting siRNA as control (Thermo Scientific/Dharmacon; D-001810-01-05).

### Oligonucleotide assay for Tdp1 activity

Tdp1 enzymatic activity was determined by cleavage of an artificial substrate as described^28^. Briefly, the assay was run at room temperature in a 96-well plate with a final volume of 100 µL per well. A final concentration of 50 nM DNA was used (5′-/56-TAMN/AGG ATC TAA AAG ACT T/3BHQ_1/-3′). Kinetic analysis was performed using a Varioskan plate reader (Thermo Fisher Scientific) at Ex557/Em582 for the TAMRA fluorophore. To establish Tdp1 cleavage activity, 20 reads were recorded at a kinetic interval of 45 seconds per read.

### Homogeneous caspase assay for apoptotic cells

The activity of caspases 3 and 7, which play key effector roles in mammalian cell apoptosis, was measured using the Apo-ONE Homogeneous Caspase-3/7 kit according to the manufacturer’s protocol (Promega). Briefly, 5 × 10^3^ cells were plated per well of a 96-well plate, cultured for 24 hours and treated as described. Fluorescence of the cleaved caspase 3 and 7 substrate was measured at 520 nm using the Wallac VICTOR2 Multilabel Plate Reader (Beckman-Coulter).

### TUNEL assay for apoptotic cells

Briefly, 5 × 10^3^ cells were seeded per well in 6-well plates, and following the described treatments, apoptotic cells were visualized by colorimetric labeling of free 3′OH DNA termini using the Apoptag Plus Peroxidase *In Situ* Apoptosis Detection Kit according to the manufacturer’s protocol (EMD Millipore). 150 cells were counted per sample group. The number of stained nuclei among 150 cells was determined for three independent replicates to quantify apoptotic cells.

### Site-directed mutagenesis of Tdp1

The p.S81A mutation was introduced into *pcDNA3*.*1*(*−*)*•TDP1*^*WT*^ by site-directed mutagenesis according to the manufacturer’s protocol using the QuikChange II XL site-directed mutagenesis kit (Agilent Technologies). *Tdp1*^−/−^ MEFs were transfected with recombinant WT or mutant Tdp1 using Lipofectamine 3000 (Invitrogen). The sequences of the oligonucleotides used are listed in Supplementary Table 2. The mutant vector was validated by Sanger sequencing (Macrogen).

### Statistical analysis

Microsoft Excel was used to compute the group means and SDs for all treatment and control groups. Statistical significance between individual groups was established by the Student t test and a P value of less than 0.05 was judged significant. The Bonferroni correction was applied to all comparisons of replicate means for the duration of the experiments.

## Results

### H_2_O_2_ treatment of cells causes Tdp1 to enter the mitochondria

**Figure 1:**
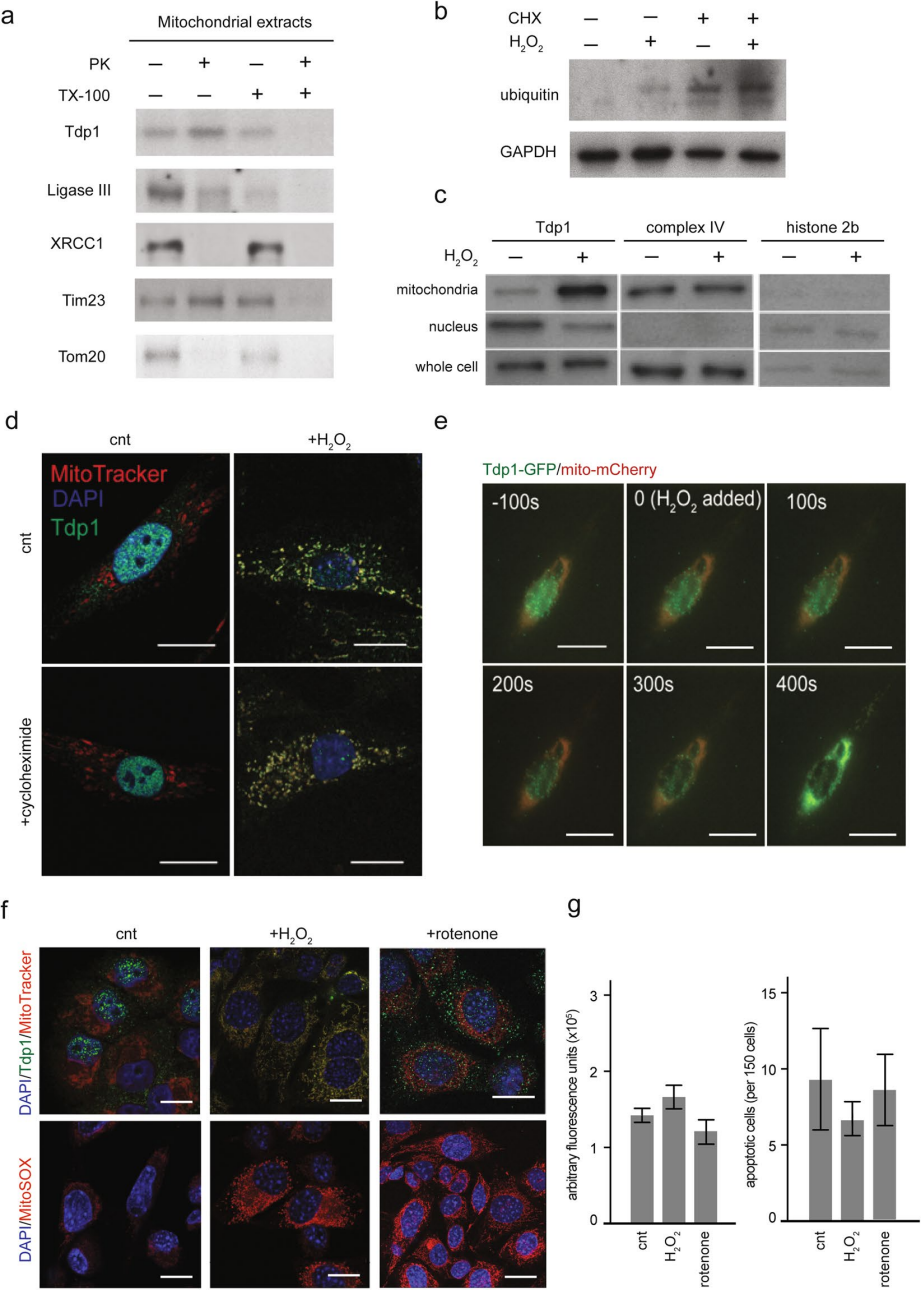
Tdp1 translocates from the nucleus into the mitochondrial matrix following H_2_O_2_ stress. (**a**) Immunoblot showing susceptibility of mitochondrial Tdp1 to proteinase K digestion in the presence and absence of the detergent Triton X-100 (TX-100). Human fibroblasts were treated with 1 µM H_2_O_2_ for 1 hour and fractionated to collect mitochondria. Mitochondrial fractions were subsequently treated with 5 µg/mL proteinase K and/or 0.25% Triton X-100. Tim23 and Tom20 are components of the inner and outer mitochondrial membranes, respectively. (**b**) Immunoblot of cytoplasmic extracts of cultured human fibroblasts in the absence or presence of treatment with 10 µM cycloheximide (CHX) and/or 1 µM H_2_O_2_ for 1 hour. GAPDH is a loading control. Indicative of inhibition of protein synthesis, the treatment with CHX caused accumulation of ubiquitylated proteins. (**c**) Immunoblot of subcellular lysates from cultured human fibroblasts treated with 10 µM cycloheximide showing movement of Tdp1 from the nucleus to the mitochondria in response to treatment with 1 µM H_2_O_2_ for 1 hour. Complex IV and histone 2b are loading controls for the mitochondria and the nucleus. (**d**) Representative immunofluorescent images showing distribution of Tdp1 within human fibroblasts in the absence or presence of 10 µM cycloheximide and/or 1 µM H_2_O_2_ treatment for 1 hour. (**e**) Live cell imaging of the distribution of Tdp1-GFP and mitochondria-targeted mCherry within cultured human fibroblasts during treatment with 1 µM H_2_O_2_. Cells were photographed every 100 seconds. (**f**) Representative immunofluorescent images showing distribution of Tdp1 and mitochondrial superoxide levels within human fibroblasts in the presence or absence of 1 µM H_2_O_2_ or 200 nM rotenone treatment for 1 hour. (**g**) Graphs showing levels of caspase-3/7 activity (left) and DNA fragmentation (right, TUNEL assay) detected in three independent experiments with human fibroblasts cultured in the absence or presence of 1 µM H_2_O_2_ or 200 nM rotenone for 1 hour. Error bars represent one standard deviation. Immunoblots are displayed in cropped format. Scale bar = 10 µm.

In a previous study, we found that the colocalization of Tdp1 with mitochondria in MEFs and human dermal fibroblasts is enhanced upon treatment with H_2_O_2_^10^. We treated both human dermal fibroblasts and MEFs with varying concentrations of H_2_O_2_ and found that a non-lethal dose of 1 µM H_2_O_2_ for 24 hours enhances colocalization of Tdp1 and mitochondria (Supplementary Figure 1a and b). To determine whether Tdp1 was associating with mitochondria or entering the mitochondrial matrix, we treated mitochondria purified from H_2_O_2_-exposed human dermal fibroblasts with proteinase K in the presence and absence of Triton X-100. Tdp1 was only digested by proteinase K following permeabilization of the mitochondrial membrane with Triton X-100 (Fig. 1a), suggesting that H_2_O_2_ treatment causes Tdp1 to enter the mitochondria. The BER protein Ligase III is observed in the mitochondrial matrix, whereas XRCC1 is not (Fig. 1a).

### Entry of Tdp1 into the mitochondria is not dependent on *de novo* protein synthesis

To determine whether Tdp1 entry into the mitochondria required *de novo* protein synthesis, we treated human dermal fibroblasts with 10 µM cycloheximide, a ribosome translocation inhibitor. Ubiquitylated proteins accumulated in cycloheximide-treated human fibroblasts, indicating blocked protein synthesis (Fig. 1b). As detected by immunoblotting and immunofluorescence, mitochondrial accumulation of Tdp1 increased upon cycloheximide and H_2_O_2_ treatment (Fig. 1c and d). Consistent with translocation of Tdp1 from the nucleus to the mitochondria, live-cell imaging of H_2_O_2_-treated human dermal fibroblasts showed loss of Tdp1 from the nucleus and increased Tdp1 accumulation within the mitochondria (Fig. 1e).

### Elevated intramitochondrial ROS is sufficient to increase mitochondrial Tdp1 accumulation

To determine whether mitochondrial Tdp1 accumulation is correlated with elevated mitochondrial superoxide levels, we stained human dermal fibroblasts with the superoxide indicator mitoSOX. Compared to untreated cells, treatment of cells with H_2_O_2_, or with 10 µM rotenone, a mitochondrial complex I inhibitor that increases levels of intramitochondrial oxidants^29^, resulted in elevated mitochondrial superoxide levels as well as mitochondrial Tdp1 accumulation (Fig. 1f). We postulate that accumulation of Tdp1 within the mitochondria did not occur because rotenone dissipates the proton motive force needed for transfer of proteins into the intermembrane space and occludes the mitochondrial outer membrane import channel^30,31^.

### Tdp1 translocation is not associated with activation of caspases 3 and 7

Although ROS stress can initiate mitochondria-dependent apoptosis^32,33^, neither rotenone nor H_2_O_2_ treatment caused an elevation in cellular caspase-3/7 activity (Fig. 1g, left panel) or nuclear DNA fragmentation (Fig. 1g, right panel). This minimizes the likelihood that Tdp1 translocation is a response to apoptotic mitochondrial signaling.

### Mitochondrial Tdp1 interacts with BER constituents and maintains mtDNA integrity

**Figure 2:**
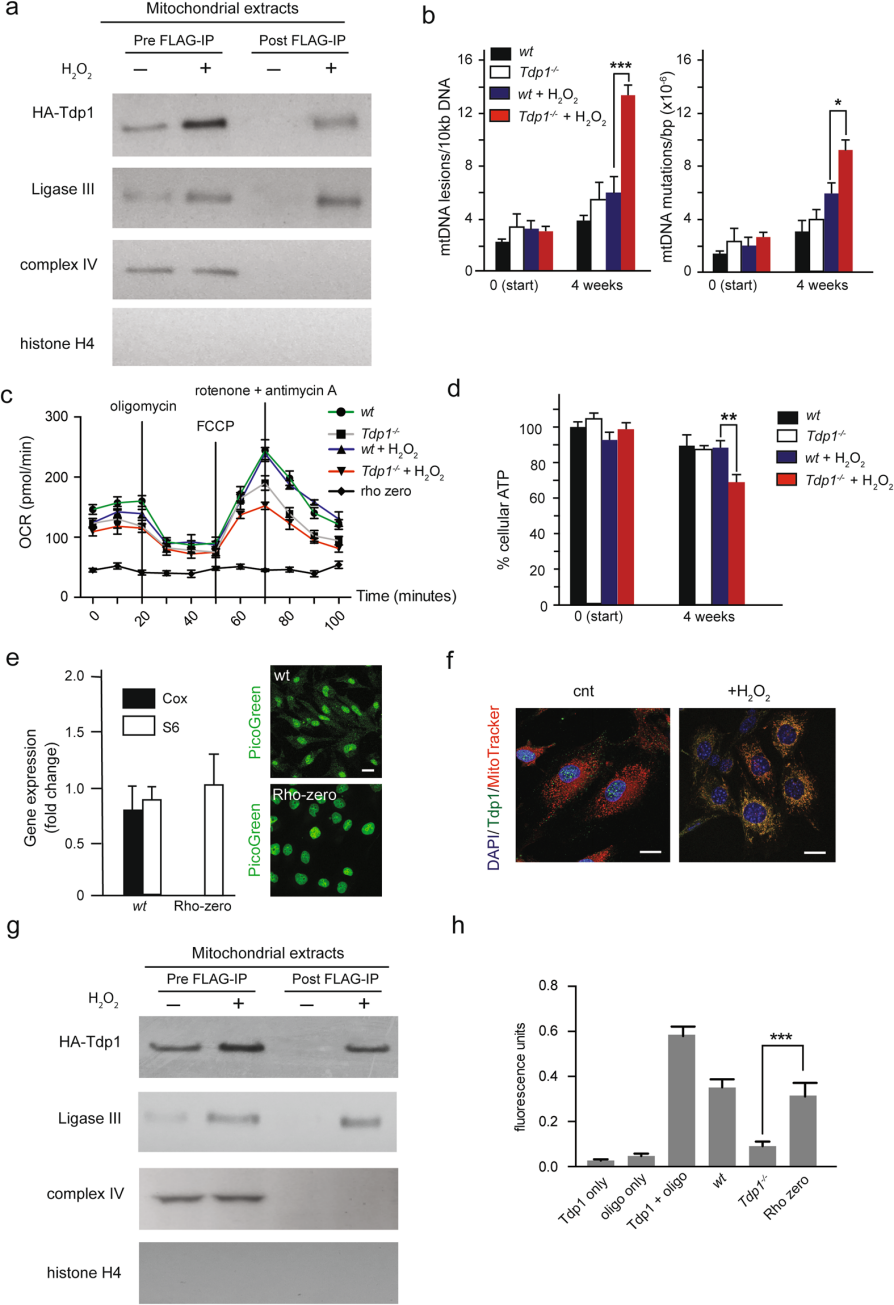
Tdp1 interacts with DNA repair proteins in the mitochondria and maintains mtDNA integrity. (**a**) Immunoblot of FLAG-HA-tagged Tdp1 immunoprecipitates from mitochondrial extracts of human fibroblasts showing co-precipitation with Ligase III. Human fibroblasts were treated with 1 µM H_2_O_2_ for 1 hour prior to purification of the mitochondria. The purified mitochondria were digested with proteinase K to remove proteins co-purifying with but external to the mitochondria. Immunoprecipitation (IP) was performed with anti-FLAG. Immunoprecipitated Tdp1 was detected using an anti-HA antibody. (**b**) mtDNA lesions (left) and mtDNA *Taq1* site mutations (right) before and following culture of MEFs with 1 µM H_2_O_2_ for 4 weeks. (**c**) Oxygen consumption rate in mitochondria of *wt* and *Tdp1*^−/−^ MEFs after 4 weeks of 1 µM H_2_O_2_ treatment. The Rho-zero cell line was used as a negative control. (**d**) ATP production in MEFs subjected to 4 weeks of 1 µM H_2_O_2_ treatment. (**e**) Left panel: normalized mitochondria-encoded cytochrome c oxidase II (Cox) and nuclear-encoded S6 ribosomal (S6) RNA levels as measured by qRT-PCR in *wt* and Rho-zero MEFs. Cox RNA levels were normalized to S6 RNA levels. Cox and S6 were used to determine the presence of transcribed mitochondrial and nuclear DNA in *wt* and Rho-zero MEFs. Right panel: picogreen staining of MEFs untreated (*wt*) or treated (Rho-zero) with ethidium bromide. Note the absence of cytoplasmic staining in Rho-zero MEFs indicating the absence of mtDNA. (**f**) Immunofluorescent staining of Tdp1 in Rho-zero MEFs with or without 1 µM H_2_O_2_ treatment for 1 hour. (**g**) Immunoblot of FLAG-HA-tagged Tdp1 immunoprecipitates from Rho-zero MEF mitochondria showing co-precipitation of Ligase III. Human fibroblasts were treated with 1 µM H_2_O_2_ for 1 hour prior to IP with anti-FLAG. Immunoprecipitated Tdp1 was detected using an anti-HA antibody. (**h**) Comparison of recombinant Tdp1 activity to Tdp1 activity in mitochondrial lysates of *wt*, *Tdp1*^−/−^, and Rho-zero MEFs after treatment with 1 µM H_2_O_2_ for 1 hour. Tdp1 activity was measured in three independent experiments; activity was detected by fluorescence following Tdp1 cleaving a quencher off a synthesized oligonucleotide substrate. The first bar shows fluorescence for purified recombinant Tdp1 without the oligonucleotide substrate; the second bar shows fluorescence for the oligonucleotide substrate without Tdp1 or mitochondrial extract; the third bar shows fluorescence for the oligonucleotide substrate with recombinant Tdp1; the fourth bar shows fluorescence for the oligonucleotide substrate with mitochondrial extracts derived from *wt* MEFs; the fifth bar shows fluorescence for the oligonucleotide substrate with mitochondrial extracts derived from *Tdp1*^−/−^ MEFs; the sixth bar shows fluorescence for the oligonucleotide substrate with mitochondrial extracts derived from Rho-zero MEFs. The oligonucleotide substrate and the purified recombinant N-terminally His-tagged Tdp1 used in this experiment were previously described^28^. *P < 0.05, **P < 0.01; ***P < 0.001. Error bars represent one standard deviation for three independent experiments. Immunoblots are displayed in cropped format. Scale bar = 10 µm.

We hypothesized that Tdp1 interacts with mtDNA repair proteins to maintain mtDNA integrity. To test for interaction with DNA repair proteins, we expressed FLAG-HA-tagged Tdp1 in human dermal fibroblasts and immunoprecipitated the tagged Tdp1 from purified mitochondria following treatment of the cells with H_2_O_2_. To remove proteins co-purifying with but external to the mitochondria, the purified mitochondria were digested with proteinase K. Following inactivation of the proteinase K with PMSF, the mitochondria were washed and then lysed. The lysates were used for anti-FLAG immunoprecipitation of the FLAG-HA-tagged Tdp1. By immunoblotting, we detected co-precipitation of Ligase III with Tdp1 (Fig. 2a), consistent with Tdp1 being a component of the mitochondrial BER complex^34,35^.

To determine if Tdp1 deficiency compromised mtDNA integrity, we harvested MEFs from *Tdp1*^−/−^ mice and assessed the level of polymerase-impeding lesions, random mutations, and deletions within the mtDNA^36–38^. As assessed by interference with *Taq* polymerase extension, both *wt* and *Tdp1*^−/−^ cells had comparable amounts of baseline lesions (Fig. 2b, left panel). After 4 weeks of culture, *Tdp1*^−/−^ MEFs harbored approximately 30% more mtDNA lesions than *wt* MEFs (Fig. 2b, left panel), and after 4 weeks of culture in the presence of 1 µM H_2_O_2_, *Tdp1*^−/−^ MEFs had 200% more mtDNA lesions than *wt* MEFs (Fig. 2b, left panel). To assess accumulation of mtDNA point mutations, we digested the mtDNA with *Taq1* and amplified the mtDNA using primers hybridizing to sequences flanking the *Taq1* recognition sites. Because mutations of the *Taq1* recognition sites block *Taq1* digestion, the amount of PCR product is proportional to the abundance of *Taq1* site mutations. This showed that *Tdp1*^−/−^ and *wt* MEFs harbored similar quantities of mtDNA mutations at baseline. However, after culturing in the presence of 1 µM H_2_O_2_ for 4 weeks, *Tdp1*^−/−^ cells had 40% more mtDNA point mutations than *wt* cells (Fig. 2b, right panel).

Because dysfunctional mtDNA repair and incomplete resolution of DNA breaks during mtDNA replication are thought to cause the formation of deleted mtDNA products and give rise to mitochondrial dysfunction^39–41^, we checked if the absence of Tdp1 favors the accumulation of mtDNA deletions. Using mtDNA extracted from *wt*, *Tdp1*^−/−^, and *PolG* null MEFs cultured in the presence or absence of 1 µM H_2_O_2_ for 4 weeks, we quantified two common mitochondrial deletion products (D1 and D13) as previously described^37^. Compared to *wt* MEFs, the *Tdp1*^−/−^ MEFs did not have markedly more of these mtDNA deletions under baseline conditions or when treated with H_2_O_2_ (data not shown).

### Tdp1 deficiency impairs mitochondrial respiration

To test whether the increase in mtDNA lesions and mutations correlated with mitochondrial respiratory dysfunction, we performed a Seahorse assay on mitochondria isolated from *wt* and *Tdp1*^−/−^ MEFs. At baseline, the *wt* and *Tdp1*^−/−^ MEF mitochondria had no significant difference in oxygen consumption rate, whereas following 4 weeks of treatment with 1 µM H_2_O_2_, *Tdp1*^−/−^ MEF mitochondria consumed 30% less oxygen than *wt* MEF mitochondria (Fig. 2c). Similarly, despite being comparable at baseline, ATP production from *Tdp1*^−/−^ MEF mitochondria was 25% less than from *wt* MEF mitochondria after 4 weeks of treatment with 1 µM H_2_O_2_ (Fig. 2d).

### Tdp1 translocates into mitochondria devoid of mtDNA

The above data define a role for Tdp1 in maintenance of mtDNA and suggest that mtDNA stress is a possible signal for Tdp1 translocation into the mitochondria. To test this, we generated Rho-zero MEFs, cells devoid of mtDNA by treatment with ethidium bromide, and validated the absence of mtDNA by loss of expression of the mtDNA-encoded complex IV subunit by qRT-PCR and by loss of mtDNA staining with PicoGreen (Fig. 2e). H_2_O_2_ treatment of the Rho-zero MEFs caused translocation of Tdp1 to the mitochondria similar to that observed in *wt* MEFs (Fig. 2f). In the mitochondria of Rho-zero cells, FLAG-HA-tagged Tdp1 formed a complex with Ligase III (Fig. 2g), and the intramitochondrial Tdp1 was enzymatically active as judged by cleavage of a 3′-quencher from a fluorescent oligonucleotide^28^ (Fig. 2h). Tdp1 translocation into the mitochondria is thus not mediated by mtDNA.

### *S*. *cerevisiae* screen identifies P38, ERK, the TIM/TOM complex and Mia40 as contributors to H_2_O_2_-dependent translocation of Tdp1 into the mitochondria

**Figure 3:**
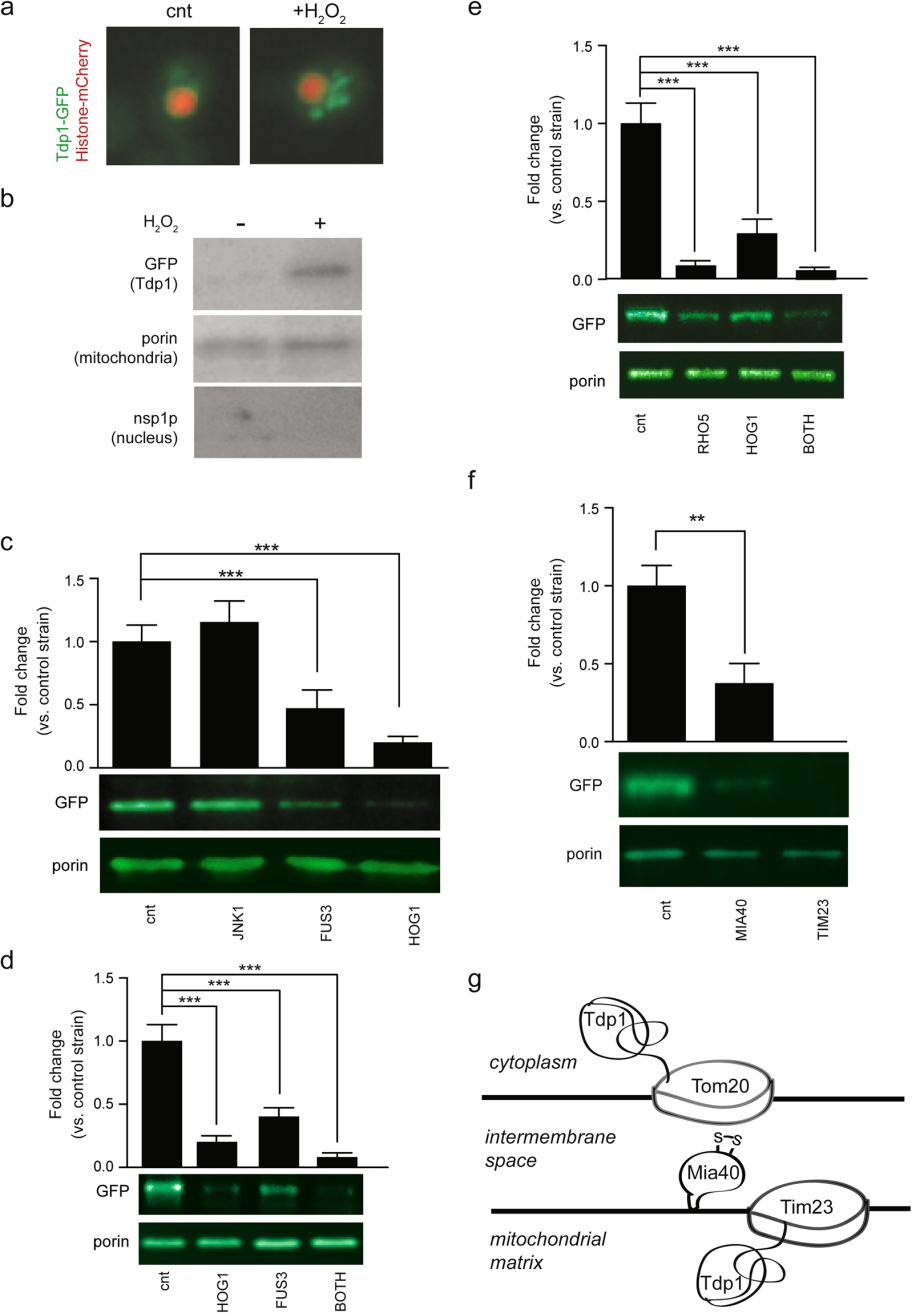
Tdp1 entry into *S*. *cerevisiae* mitochondria under H_2_O_2_ stress is facilitated by MAP kinases and the canonical mitochondrial import pathway. (**a**) Yeast expressing Tdp1-GFP and Histone-mCherry show cytoplasmic accumulation of Tdp1-GFP following H_2_O_2_ treatment. The images were taken with or without treatment with 5 µM H_2_O_2_ for 1 hour. *S*. *cerevisiae* require higher H_2_O_2_ concentrations than cultured mammalian fibroblasts for translocation of Tdp1 from the nucleus to the cytoplasm; 5 µM H_2_O_2_ was chosen based on a dose response curve (1, 5, 10, 50, 250 µM) for cytoplasmic localization of Tdp1 at 1 hour. (**b**) Immunoblot showing accumulation of Tdp1-GFP in lysates from purified mitochondria after treatment of yeast with 5 µM H_2_O_2_ for 1 hour. Porin is a mitochondrial protein and nsp1p is a nuclear protein. (**c**) Upper panel: Quantification of mitochondrial Tdp1-GFP following treatment of yeast control and knockouts JNK1, FUS3 and HOG1 with 5 µM H_2_O_2_ for 1 hour. Tdp1-GFP levels were normalized to porin levels. JNK1, FUS3 and HOG1 are yeast orthologs of human JNK, ERK1 and P38. Lower panel: Immunoblot of lysates from purified mitochondria of the corresponding yeast strains. (**d**) Upper panel: Quantification of mitochondrial Tdp1-GFP following treatment of yeast control and knockouts FUS3, HOG1 and FUS3 + HOG1 (BOTH) with 5 µM H_2_O_2_ for 1 hour. Tdp1-GFP levels were normalized to porin levels. Lower panel: Immunoblot of lysates from purified mitochondria of corresponding yeast strains. (**e**) Upper panel: Quantification of mitochondrial Tdp1-GFP following treatment of yeast control and knockouts RHO5, HOG1 and RHO5 + HOG1 (BOTH) with 5 µM H_2_O_2_ for 1 hour. Tdp1-GFP levels were normalized to porin levels. Lower panel: Immunoblot of lysates from purified mitochondria of the corresponding yeast strains. RHO5 is the yeast ortholog of human RAS. (**f**) Upper panel: Quantification of mitochondrial Tdp1-GFP following treatment of yeast control and knockouts MIA40 and TIM23 with 5 µM H_2_O_2_ for 1 hour. Tdp1-GFP levels were normalized to porin levels. Immunoblot of lysates from purified mitochondria of corresponding yeast strains. (**g**) Schematic drawing illustrating a possible mechanism for Tdp1 transport across the mitochondrial double-membrane. *P* < 0.05; **P < 0.01; ***P < 0.001. Error bars represent one standard deviation for three independent experiments.

To determine if intramitochondrial ROS is the origin of the signal for translocation of Tdp1, we used *S*. *cerevisiae* to screen for factors participating in the H_2_O_2_-dependent translocation of Tdp1 to the mitochondrial matrix. After 5 µM H_2_O_2_ treatment, we observed Tdp1 expression in the cytoplasm of yeast by fluorescence microscopy and in the mitochondrial fraction by immunoblotting (Fig. 3a and b). H_2_O_2_ exposure is a known activator of Rho5, mitogen-activated protein (MAP) kinase P38, ERK1 and JNK1^42^. P38 and ERK1 are respectively orthologs of yeast HOG1 and FUS3. Knocking out each of *HOG1* and *FUS3*, but not *JNK1*, reduced Tdp1 translocation into the mitochondria by 70–80% at 1 hour following exposure to 5 µM H_2_O_2_ (Fig. 3c). Indicative of HOG1 and FUS3 redundantly regulating Tdp1 translocation, the double-knockout of *HOG1* and *FUS3* inhibited Tdp1 translocation more than the individual knockouts (95% vs. 70–80%, Fig. 3d). Suggesting that Rho5 mediates this activation of HOG1 and FUS3, knockout of *Rho5* alone and double-knockout of *RHO5* and *HOG1* reduced Tdp1 translocation into the mitochondria by 95% at 1 hour following exposure to 5 µM H_2_O_2_ (Fig. 3e).

Knockout of the mitochondrial intermembrane transporter *MIA40*, a redox-responsive protein^43^, reduced H_2_O_2_-dependent translocation of Tdp1 by 85% (Fig. 3f). Knockout of *TIM23*, a subunit of the inner membrane translocase of the canonical mitochondrial protein import complex completely abrogated Tdp1 import (Fig. 3f). These results indicate that Tdp1 transport proceeds through the canonical TIM-TOM complexes and is chaperoned through the intermembrane space by Mia40 (Fig. 3g).

### siRNA knockdown of P38, ERK1, and TIM23 impedes H_2_O_2_-dependent translocation of Tdp1 into the mitochondria of human dermal fibroblasts

**Figure 4:**
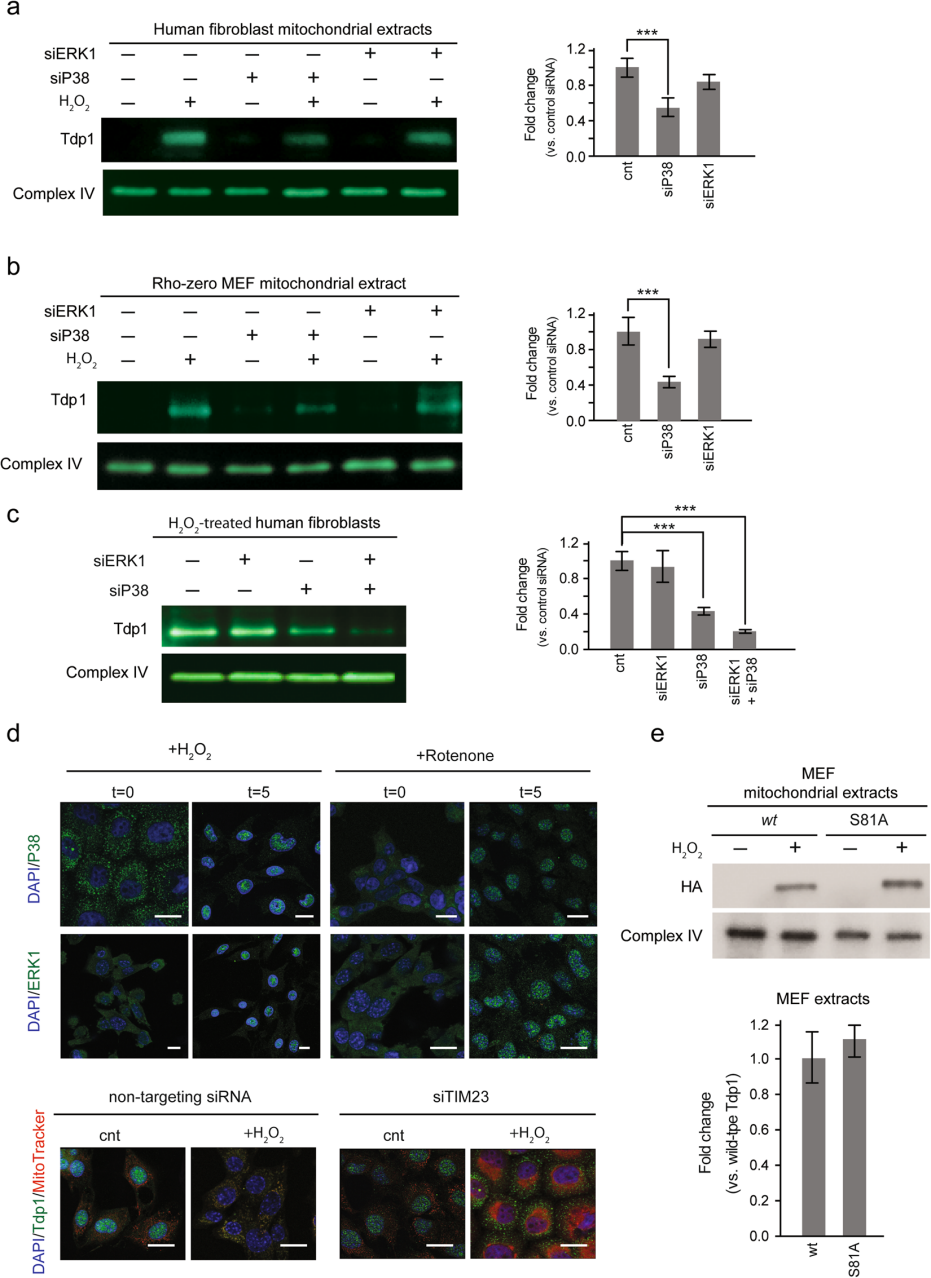
Conserved pathways facilitate Tdp1 entry into human and mouse mitochondria following H_2_O_2_ stress. (**a**) Left panel: Immunoblot of human fibroblast mitochondrial lysates without or with knockdown of ERK1 and P38 and in the absence or presence of 1 µM H_2_O_2_ for 1 hour. Right panel: Graph of mitochondrial Tdp1 levels across three independent experiments following treatment with 1 µM H_2_O_2_ for 1 hour. Tdp1 levels were normalized to complex IV and fold changes are shown relative to levels in cells treated with nontargeting siRNA. (**b**) Left panel: Immunoblot of Rho-zero MEF mitochondrial lysates without or with knockdown of ERK1 and P38 and in the absence or presence of H_2_O_2_ treatment. Mitochondrial complex IV was used as a loading control. Right panel: Graph of mitochondrial Tdp1 levels across three independent experiments following treatment with 1 µM H_2_O_2_ for 1 hour. (**c**) Left panel: Immunoblot of human fibroblast mitochondrial lysates without or with knockdown of ERK1 and P38 and in the presence of H_2_O_2_ treatment. Right panel: Graph of mitochondrial Tdp1 levels across three independent experiments following treatment with 1 µM H_2_O_2_ for 1 hour. (**d**) Upper panel: Immunofluorescent staining of P38 and ERK1 in human fibroblasts before and after 5 minutes with 1 µM H_2_O_2_ or 200 nM rotenone treatment. Lower panel: Immunofluorescent staining of Tdp1 in human fibroblasts treated with non-targeting siRNA or TIM23 siRNA with or without 1 µM H_2_O_2_ for 1 hour. (**e**) Upper panel: Immunoblot of lysates from purified mitochondria of cultured MEFs expressing *wt* or S81A HA-tagged Tdp1 cDNA in the presence or absence of treatment with 1 µM H_2_O_2_ for 1 hour. Lower panel: Graph of fold change in mitochondrial Tdp1 across three independent experiments following treatment with 1 µM H_2_O_2_ for 1 hour. Tdp1 levels were normalized to complex IV levels. Error bars represent one standard deviation. *P < 0.05; **P < 0.01; ***P < 0.001. Mitochondrial complex IV was used as a loading control. Scale bar = 10 µm.

Similar to *S*. *cerevisiae*, exposure of cultured human dermal fibroblasts to H_2_O_2_ activates mitogen-activated protein (MAP) kinases P38, ERK1 and JNK (the respective human orthologs of HOG1, FUS3, JNK1)^44^. We found that exposure of human dermal fibroblasts to 1 µM H_2_O_2_ for 1 hour promoted the maximal phosphorylation of P38 and ERK1 but did not promote the phosphorylation of JNK (Supplementary Figure 1c). Given that Tdp1 translocated into the mitochondria within 6–7 minutes at 1 µM H_2_O_2_ (Fig. 1e), we focused on P38 and ERK1 as mediators of H_2_O_2_-dependent mitochondrial Tdp1 translocation. Using siRNA, an 80% knockdown of P38 resulted in a 50% reduction in H_2_O_2_-mediated mitochondrial Tdp1 translocation, and a 70% knockdown of ERK1 resulted in a 20% reduction in H_2_O_2_-dependent mitochondrial Tdp1 translocation (Fig. 4a and Supplementary Figure 1d). Concurrent 80% knockdown of P38 and ERK1 resulted in an 80% reduction in H_2_O_2_-dependent mitochondrial Tdp1 translocation (Fig. 4c). Similarly, knockdown of P38 or ERK1 in Rho-zero MEFs also impaired H_2_O_2_-dependent transport of Tdp1 into the mitochondria (Fig. 4b).

The decrease in mitochondrial Tdp1 after P38 and ERK1 knockdown is due to retention of nuclear Tdp1 (Supplementary Figure 2a and b). Confirming that intramitochondrial ROS generated by addition of 1 µM rotenone activated the same pathways, 80% knockdown of P38 or 75% knockdown of ERK1 increased the retention of Tdp1 in the nucleus (Supplementary Figure 2c and d).

P38 and ERK1 translocate from the cytoplasm into the nucleus upon phosphorylation and activate nuclear substrates^45,46^. Within 5 minutes of adding 1 µM H_2_O_2_ or 1 µM rotenone to human dermal fibroblasts, we detected increased nuclear P38 and ERK1 (Fig. 4d). This timing is consistent with Tdp1 translocation out of the nucleus and into the mitochondria (Fig. 1e) and suggests that P38 and ERK1 activate nuclear targets facilitating Tdp1 translocation from the nucleus to the mitochondria.

Consistent with the yeast studies, siRNA knockdown of TIM23 in human dermal fibroblasts reduced Tdp1 translocation into the mitochondria and resulted in Tdp1 aggregation in the cytoplasm (Fig. 4d). Mitochondrial import of Tdp1 via the canonical TIM-TOM pathway is thus conserved between human and yeast cells.

Tdp1 does not contain a canonical mitochondria-targeting presequence identifiable by mitochondrial targeting sequence predictors TargetP, MitoProt and MitoFates (data not shown). Additionally, serial truncations from either the N- or C-terminus of Tdp1 did not identify a distinct mitochondrial-targeting signal; rather they identified contributions from multiple regions of the Tdp1 protein to Tdp1 mitochondrial entry (data not shown). In addition, site-directed mutagenesis of Ser81, a phosphorylation site in Tdp1, showed that changing serine 81 to alanine was insufficient to block H_2_O_2_-dependent translocation of Tdp1 into the mitochondria (Fig. 4e)^47^.

### siRNA knockdown of Tdp1, P38, ERK1, and TIM23 increases H_2_O_2_-dependent mtDNA mutations in human dermal fibroblasts

Similar to our observation in *Tdp1*^−/−^ MEFs, siRNA knockdown of Tdp1 in human fibroblasts led to an increase in random mtDNA mutations after treatment with H_2_O_2_ (Supplementary Figure 2e). Because P38, ERK1 and TIM23 enable the translocation of Tdp1 into mitochondria, we hypothesized that transient knockdown of any of these proteins increases random mtDNA mutations in human fibroblasts treated with H_2_O_2_. Indeed, knocking down P38, ERK1 and TIM23 in the presence of H_2_O_2_ led to an increase in random mutations by 45%, 20% and 65%, respectively, over the course of 96 hours. (Supplementary Figure 2e). Further study is required to determine if inhibition of these pathways contributes to the increase in mtDNA mutations by impeding transport of other DNA repair factors besides Tdp1.

### *Tdp1*^−/−^ mice accumulate mutations in mtDNA and have impaired mitochondrial respiration

**Figure 5:**
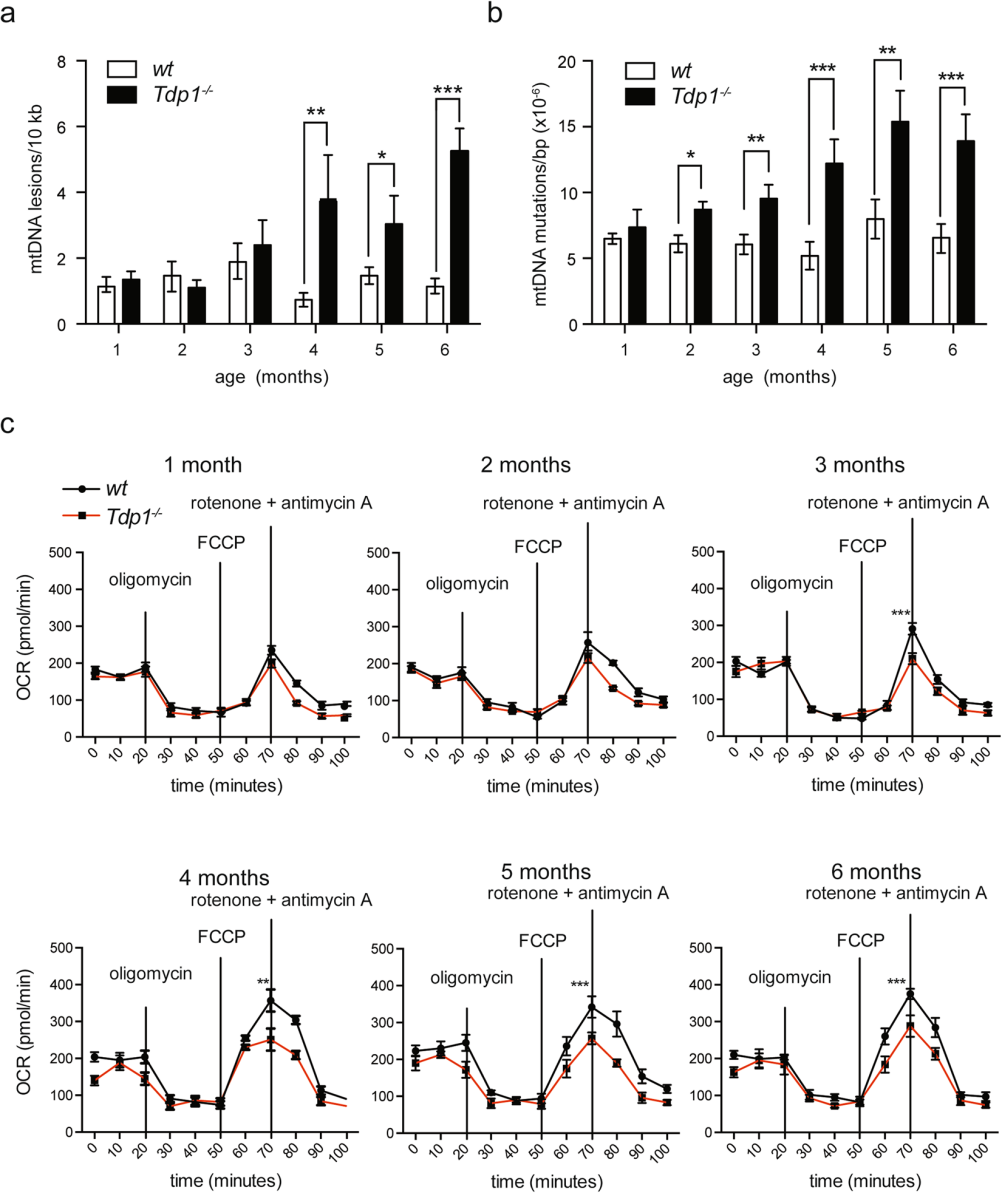
Tdp1 maintains mtDNA integrity and respiratory function in mouse mitochondria. (**a**) Quantification of mtDNA lesions detected by qPCR in isolated skeletal muscle mitochondria of 8 *wt* and 8 *Tdp1*^−/−^ mice at ages 1 through 6 months. Each bar is the mean of three independent experiments, and error bars represent one standard deviation. (**b**) Quantification of *Taq1* cut site mutations detected by qPCR in pooled mtDNA isolated from skeletal muscle mitochondria of 8 *wt* and 8 *Tdp1*^−/−^ mice at ages 1 through 6 months. Each bar is the mean of three inde-pendent experiments, and error bars represent one standard deviation. (**c**) Analysis of mitochondrial oxygen consumption rate in cultured *wt* and *Tdp1*^−/−^ primary myoblasts harvested and pooled from 8 mice at the age of 1 through 6 months. 2 µM oligomycin was used to inhibit ATP production. 2 µM of the ionophore FCCP was used to uncouple the electron transport chain, allowing maximal respiration. 500 nM rotenone and antimycin A were used to inhibit ATP synthesis. Each bar is the mean of three independent experiments, and error bars represent one standard deviation. *P < 0.05, **P < 0.01; ***P < 0.001.

Given that long-term culture of *Tdp1*^−/−^ MEFs in low dose H_2_O_2_ is detrimental to cultured cells, we examined the mtDNA integrity and the mitochondrial respiratory function of skeletal muscle from 1-month-old to 6-month-old *Tdp1*^−/−^ mice. Analysis of polymerase-interrupting lesions in mtDNA showed a 70% to 400% increase in these lesions in 4-, 5- and 6-month-old *Tdp1*^−/−^ mice (Fig. 5a). Quantification of mutations that inhibited *Taq1* digestion showed that myoblast mtDNA from 1-month-old *Tdp1*^−/−^ mice and *wt* mice had a comparable mutation burden (Fig. 5a), whereas myoblast mtDNA from 2-, 3-, 4-, 5- and 6-month-old *Tdp1*^−/−^ mice had mtDNA mutation loads 20%–120% higher than *wt* mice myoblast mtDNA (Fig. 5b).

Since mutations in mtDNA-encoded genes can alter mitochondrial function, we examined mitochondrial respiration using myoblasts isolated from skeletal muscle. Comparative Seahorse analysis of myoblast cultures from *wt* and *Tdp1*^−/−^ mice showed that *Tdp1*^−/−^ myoblasts isolated from 3-, 4-, 5-, and 6-month-old mice were unable to achieve full respiratory potential; that is, uncoupling of the electron transport chain by FCCP treatment detected a 30–60% reduction in oxygen consumption rate in *Tdp1*^−/−^ myoblasts compared to *wt* myoblasts (Fig. 5c).

## Discussion

Since the discovery of Tdp1 as the main 3′-DNA phosphodiesterase, the biochemical activity of Tdp1 has been well characterized^3,5,6,35,48–53^. We herein report the first delineation of Tdp1 transport into the mitochondria and its significance for mitochondrial function. Within *S*. *cerevisiae*, mouse and human cells, intramitochondrial ROS induce Tdp1 translocation from the nucleus to the mitochondria via P38- and ERK1-dependent mechanisms. This translocation is independent of new protein synthesis and of mtDNA. Our findings also show that Tdp1 interacts with BER protein Ligase III within mitochondria and that cultured cells and tissues deficient for Tdp1 have more mtDNA lesions, accumulate mtDNA mutations, and have impaired mitochondrial respiration.

Given that exogenous rotenone stimulated the transport of Tdp1 out of the nucleus via the P38 and ERK1 signaling cascades, intramitochondrial ROS appears sufficient for Tdp1 translocation from the nucleus to the mitochondria. Unlike H_2_O_2_, however, rotenone treatment did not result in the entry of Tdp1 into the mitochondrial matrix. Two factors account for this failure. First, inhibition of complex I by rotenone blocks electron transfer between complex I and co-enzyme Q and prevents the concurrent transfer of hydrogen ions out of the matrix into the intermembrane space; this decreases the proton motive force driving transfer of proteins into the intermembrane space^30^. Second, rotenone impedes mitochondrial import by occluding the mitochondrial outer membrane import channel^31^.

Although intramitochondrial ROS likely signals translocation of Tdp1 from the nucleus to the mitochondrial matrix, the mediating MAP kinase P38 and ERK1 reside in the cytoplasm. This implies that either the intramitochondrial ROS or a signal from the intramitochondrial ROS crosses the mitochondrial membranes to activate these signaling cascades. A possible mediator is H_2_O_2_, a membrane-permeable signaling molecule produced by NADPH oxidases (NOX), cyclooxygenases and lipooxygenases^54^. H_2_O_2_ might therefore be the messenger activating the extra-mitochondrial pathways of stress-activated protein kinases; it also inhibits phosphatases that attenuate signaling of cell survival pathways, and in the nucleus it activates transcription factors by oxidation of cysteine residues^55^. Further studies are required to determine if H_2_O_2_ is the mitochondrial to cytoplasmic messenger mediating Tdp1 translocation.

The mechanisms by which the P38 and ERK1 signaling cascades trigger Tdp1 translocation from the nucleus to the mitochondrial matrix are unknown. The rapid translocation of P38 and ERK1 from the cytoplasm to the nucleus following generation of intramitochondrial ROS suggests that Tdp1 or a nuclear chaperone of Tdp1 is the target of P38 and ERK1. Although phosphorylation of Tdp1 serine 81 activates repair of topoisomerase-I mediated DNA damage, promotes Tdp1 interaction with Ligase III, and enhances the stability of Tdp1 *in vitro*^34,47^, it is not required for translocation of Tdp1. This suggests that, if Tdp1 is a direct target of P38 and ERK1, then other serines or threonines are phosphorylated.

A surprising observation was that mtDNA was unnecessary for translocation of Tdp1 into the mitochondria or its incorporation into the BER complex. These findings are consistent with a prior study showing that components of the BER complex reside in the mitochondria of Rho-zero cells^56^. We conclude that Tdp1 is recruited by and incorporated into the mitochondrial BER complex under conditions predisposing to DNA damage, i.e., reactive oxygen species, and not by the DNA damage itself. We also hypothesize that the increase in and the coprecipitation of Tdp1 and Ligase III from the mitochondrial matrix of Rho-zero cells after H_2_O_2_ treatment indicates that Tdp1 interacts in the mitochondria with Ligase III, a component of the BER complex^9^ and that, independent of DNA damage, it participates in the formation of the mitochondrial BER complex under conditions predisposing to DNA damage, i.e., reactive oxygen species.

Previous studies have shown that oxidative base lesions induce the formation of stalled topoisomerase I-DNA cleavage complexes within the nucleus^57^ and that Tdp1 participates in repair of such complexes^6^. Knowing that there is a mitochondrial topoisomerase I^58^, we postulate that, similar to the nucleus, oxidative agents in the mitochondrial matrix cause formation of 8-oxoguanine lesions and subsequently of stalled mitochondrial topoisomerase I-DNA cleavage complexes and that Tdp1 participates in the repair of these. Additionally, because the promiscuity of mitochondrial DNA polymerase γ (polG) can lead to the incorporation of chain-terminating nucleoside analogs, which are used as drugs in antiviral and anticancer therapy, mitochondrial Tdp1 might also repair blocked 3′-mtDNA ends caused by the incorporation of these nucleoside analogs^3^.

Maintenance of mtDNA is crucial because the mitochondrial genome consists almost entirely of open reading frames encoding essential proteins, rRNAs, or tRNAs. Despite the importance of mtDNA repair, little detail is known about mtDNA repair mechanisms or the transport of DNA repair proteins into the mitochondria. Approximately 1,500 nuclear-encoded proteins are transported to the human mitochondria^59^. Most of these proteins thread through mitochondrial membrane translocases coincident with protein synthesis^60^. Many proteins destined for the matrix contain matrix-targeting pre-sequences recognized by the outer and inner membrane translocase (TOM/TIM) subunits^61^, which form a channel to the matrix^60^. In the intermembrane space, chaperones guide proteins with these signal sequences^62^. Once in the matrix, the pre-sequence is cleaved by Matrix Processing Peptidase (MPP)^63^. We did not, however, identify a Tdp1 targeting pre-sequence; therefore Tdp1 classifies as a mitochondrial matrix protein without an identifiable targeting pre-sequence^64,65^.

In the absence of a targeting pre-sequence, Tdp1 mitochondrial translocation might rely on cryptic mitochondrial targeting residues. The presence of such residues usually results in truncated isoforms of the protein within the mitochondria following post-translational endoprotease processing of the protein into a translocation-active form^66,67^. This is unlikely for Tdp1 because truncated isoforms of Tdp1 were not detected by immunoblotting of mitochondrial extracts.

The p.H493R mutation in Tdp1 causes Spinocerebellar Ataxia with Axonal Neuropathy 1 (SCAN1), a neurodegenerative disease^68^. This mutation reduces the enzymatic activity of Tdp1 by 25-fold and causes accumulation of Tdp1-DNA reaction intermediates^22,69^. Our findings that skeletal muscle isolated from *Tdp1*^−/−^ mice have an increased mtDNA mutation load and that myoblast mitochondria isolated from *Tdp1*^−/−^ mice have significantly reduced respiratory capacity suggest that mitochondrial dysfunction contributes to the pathogenesis of SCAN1.

In summary, we show that cytoplasmic or mitochondrial ROS promotes the translocation of Tdp1 from the nucleus to the mitochondria of yeast, mouse and human cells and that translocation occurs independently of protein synthesis and the presence of mtDNA. Transport of nuclear Tdp1 to the mitochondria is mediated by P38 and ERK1 MAP kinase activation, and Tdp1 entry into the mitochondrial matrix is through the TIM/TOM complexes. Within the mitochondria, Tdp1 associates with the BER complex, and consistent with a role for Tdp1 in mtDNA repair, mtDNA from *Tdp1*^−/−^ mouse tissue has increased mutations and lesions. Functionally, this translates into *Tdp1*^−/−^ mouse myoblasts having impaired mitochondrial respiration.

These findings define a mitochondrial role for Tdp1, although further work is needed to shed light on mtDNA integrity and mitochondrial function in the neurons of *Tdp1*^−/−^ mice as precedent for understanding the neuropathology of SCAN1 patients. Additionally, given that several cancers have elevated Tdp1 expression^23,70,71^ and that evasion of apoptotic cell death increases tumorigenesis^72^, one might also hypothesize that the mitochondrial localization of Tdp1 favors the survival of cancer cells.

## Electronic supplementary material

Supplementary Material
