# Supplementary material for: Reactive oxygen species stress increases accumulation of tyrosyl-DNA phsosphodiesterase 1 within mitochondria
Source: Sci Rep. 2018 Mar 9;8:4304. doi: 10.1038/s41598-018-22547-8 (PMC5844879; doi:10.1038/s41598-018-22547-8)

Supplementary Material

Reactive oxygen species stress increases accumulation of tyrosyl-DNA phosphodiesterase 1 within mitochondria

Hok Khim Fam[1,2], Kunho Choi[1,2], Lauren Fougner[1,2], Chinten James Lim[1,3], *Cornelius F. Boerkoel[1,2]

[1]BC Children's Hospital Research Institute, [2]Department of Medical Genetics, [3]Department of Pediatrics, University of British Columbia, Vancouver, Canada

*Corresponding Author

Supplementary Methods

Subcellular fractionation of human and mouse cells

To purify mitochondria, confluent cultured cells from five 10 cm dishes were released using trypsin and pooled. The cells were lysed in lysis buffer A (Mitochondria Isolation Kit for Cultured Cells, Abcam) and centrifuged at 14,000 × *g* to obtain a crude mitochondrial pellet and supernatant containing cytoplasmic proteins without contaminating mitochondria. The mitochondria were purified from this crude pellet by ultracentrifugation at 80,000 × *g* for 4 hours using a step gradient of Optiprep^{TM} (60% iodixanol, AXIS-SHIELD). The step gradient was prepared using 40% (w/v), 30% (w/v) and 10% (w/v) iodixanol and the crude mitochondrial pellet was dispersed in the bottom 40% layer. Mitochondria were collected from the 30%-10% iodixanol interface and diluted 1:5 with mitochondria isolation buffer (0.25 M sucrose, 1 mM EDTA, 20 mM HEPES-NaOH, pH 7.4). This preparation was centrifuged at 14,000 × *g* for 10 minutes to obtain a mitochondrial pellet for immunoblotting. All steps were done at 4º C, and all solutions and buffers contained 1X protease inhibitor cocktail (Roche).

Protease treatment of isolated mitochondria

Isolated mitochondria were treated with 25 mg/ml proteinase K in SEM buffer (250mM sucrose, 1 mM EDTA, and 10 mM MOPS, pH 7.2) in the absence or presence of 1% Triton X-100 for 15 min at 4°C. Proteinase K was inactivated by the addition of PMSF to a final concentration of 4 mM. The samples were incubated for 10 min at 4°C. Mitochondria were pelleted and washed with SEM buffer. In the case of Triton lysates, the supernatant was used directly for SDS-PAGE analyzes.

Subcellular fractionation of yeast cells

The fractionation of yeast cells was adapted from a previous study [1]. Briefly, yeast cells were grown to mid-exponential phase in YPD and harvested by centrifugation at 3,000 × *g*. The cells were then resuspended in 100 mM tris-sulphate buffer (pH 9.4) and 10 mM DTT; the yeast cells were then treated with Zymolase 100T at a concentration of 5 μg/OD_{600} unit of cells at 30°C. Spheroplasts were then harvested by centrifugation at 1,500 × *g* at room temperature and washed twice in homogenization medium (HM; 250 mM sucrose, 10 mM HEPES (pH 7.4), 1 mM EDTA (pH 8.0) and 1 mM DTT) supplemented with complete protease inhibitor (Roche). Spheroplasts were lysed with 20 strokes of a tight-fitting Dounce homogenizer, and then the cell homogenate was centrifuged at 10,000 × *g* for 15 minutes at 4°C. The pellet was suspended in 2 mL 250 mM sucrose, 10 mM HEPES (pH 7.4) and 1 mM EDTA (pH 8.0) buffer, and layered on top of a 10 mM HEPES-buffered sucrose step gradient (2 mL, 15%; 3 mL, 25%; 3 mL, 40%; and 2 mL, 60%, respectively) and centrifuged for 1 hour at 4°C at 32,000 rpm in a SW32Ti rotor (Beckman). The visible reddish-brown mitochondria-enriched layer was collected into a 2-mL centrifuge tube.

Generation of Rho-zero MEFs

Mouse embryonic fibroblasts were treated with 100 ng/mL of ethidium bromide in DMEM (Gibco BRL Life Technologies) supplemented with 10% heat-inactivated FBS (Hyclone) and 1% antibiotic-antimycotic (Gibco BRL Life Technologies), 2.5 mM sodium pyruvate and 50 μg/mL uridine for 2 weeks. Cells were considered Rho-zero when expression of mitochondrial genes was undetectable by RT-PCR and mtDNA was undetectable by PicoGreen staining, which was performed as previously described [2]. To determine the expression of

mitochondrial (i.e., cytochrome oxidase II) and nuclear (i.e., S6 ribosomal subunit) genes, total RNA was isolated using the Qiagen RNeasy Mini Kit (Qiagen). 3 μg of RNA was reverse transcribed with qScript cDNA Supermix using a proprietary blend of random and oligo (dT) primers (Quanta Biosciences). Quantitative PCR of the cDNA was performed with the PerfeCTa SYBR Green SuperMix mix (Quanta Biosciences) using the Applied Biosystems 7500 Real-Time PCR System. Data was analyzed using the Applied Biosystems 7500 software. Primer sequences are listed in Supplementary Table 2.

Time-lapse photography of Tdp1 transport

For live-cell imaging, 3×10^5 human fibroblasts expressing Tdp1-cGFP and a mitochondria-targeted mCherry were seeded into an opaque-walled 35 mm glass-bottom cell culture dish (Willco Wells) and placed in a 37°C and 5% CO_2 environmental chamber (Precision Control Systems). Images were taken using a 96X objective with the MetaMorph 7.5 software (Molecular Devices) on an Olympus IX81 epifluorescent microscope. Images were taken once every 100 seconds and stitched together using MetaMorph (Molecular Devices).

Isolation of mouse skeletal muscle myoblasts

Mouse skeletal myoblast isolation was performed as described [3]. Briefly, the limbs of 3 mice were rinsed with 70% ethanol. The muscle tissue was dissected away from the skin and bone and placed into a tissue culture dish. The tissue was kept submerged in PBS and finely chopped with a razor blade and then triturated several times to break up larger clumps. The cells were centrifuged at 4°C, 350 × *g* for 5 minutes. Two mL of collagenase solution per gram of tissue was added to the minced tissue and incubated at 37°C for 20 minutes. The tissue mixture

was triturated every 5 minutes to mix collagenase thoroughly with the tissue. This slurry was then filtered through an 80-micron filter and the eluted cells were centrifuged for 5 minutes at 400 × *g*. The pellet was suspended in 4 mL of F-10 myoblast growth medium and plated into a 35-mm collagen-coated tissue culture plate. To enrich for myoblasts, the cells were incubated at 37°C, 5% CO_2 for several days. The F-10 culture medium was changed every 2 days. When 80% confluent, the F-10 culture medium was removed and the cells were incubated in 1X PBS for 2 minutes at 37°C. Following aspiration of the PBS, the myoblasts were selected by hitting the side of the dish firmly with the palm of the hand to allow the myoblasts to come off the bottom of the plate and leave the fibroblasts behind. The myoblasts were then plated on a collagen-coated dish for 15 minutes to allow residual fibroblasts to stick to the dish. The cells remaining in suspension after 15 minutes were transferred into a new dish; passaging was repeated until fibroblasts were removed.

Seahorse analysis of cultured cells and myoblasts

Seahorse analysis was performed as described [4]. Briefly, the plates were put in a Seahorse XF24 extracellular flux analyzer at 37°C (Agilent Technologies) for a 10-minute calibration and 3 measurement cycles to record basal cellular respiration. Oligomycin (5 µg/mL), FCCP (5 µM), and a mixture of rotenone (1 µM) plus antimycin A (2 µM) were then added sequentially to inhibit the ATP synthase, to uncouple oxidative phosphorylation, and to gauge non-mitochondrial respiration, respectively.

Measurement of ATP production in cultured cells

ATP production in cultured cells was measured using the CellTiter-Glo® Assay kit (Promega). Briefly, 1 x 10^4 cells were seeded in 100 µL of culture media, in an opaque-walled 96-well plate and placed in an incubator overnight at 37°C, 5% CO_2. The following day, 100 µL of CellTiter-Glo® reagent was added to each well. The 96-well plate was then put onto an orbital shaker for 2 minutes to lyse the cells and incubated for 10 minutes at room temperature. Luminescence was measured at 520 nm using the Wallac VICTOR2 Multilabel Plate Reader (Beckman-Coulter).

Mitochondrial random mutation assay

Mutations in the mouse mitochondrial genome were quantified as previously described [5]. Briefly, mtDNA was digested with *TaqI* for 5 hours, 100 units of *TaqI* was added every hour. The mtDNA was aliquoted at 5 ng/µL per well of a 96-well plate. PCR amplification of regions encompassing *TaqI* restriction sites was performed using the primers listed in Supplementary Table 2. Primers that did not flank a *TaqI* restriction site were used to quantify the mtDNA genomes. PCR amplification was performed as follows: step 1, 37°C for 10 minutes; step 2, 95°C for 10 minutes; step 3, 95°C for 30 seconds; step 4, 58/60°C for 1 minute; step 5, 72°C for 1.5 minutes; step 6, go to step three 44 times; step 7, 72°C for 4 minutes; step 8, melting curve from 65°C to 95°C; step 9, hold at 4°C indefinitely. PCR reactions were carried out in 25 µL reactions using the Brilliant SYBR-green real-time PCR master mix (Thermo Fisher Scientific), 10 pM of forward and reverse primers, and 1 unit of uracil DNA glycosylase.

Mitochondrial DNA lesion assay

Mitochondrial DNA lesions were quantified by long-amplicon quantitative PCR as described [6]. This method allows the assessment of polymerase-impeding lesions and breaks in

the DNA by quantitative PCR of specific long (~10 kb) and short (~100 bp) fragments of mtDNA. The relative PCR product of the long fragment is normalized to the short fragment to obtain a relative lesion frequency. The mtDNA in short fragment is approximately 100 times less likely to be damaged than the long fragment and is used as a control for mtDNA input. The Primers amplifying the short and long mtDNA regions of interest are listed in Supplementary Table 2. Briefly, the LongAMP Hot Start Taq 2x (New England Biolabs) was used to amplify mtDNA by quantitative PCR. The PCR master mix consisted of the following: nuclease-free water, LongAmp Master Mix, 10 µM primers, and 15 ng total cellular DNA template per 50 µL reaction. PCR amplification was performed as follows: step 1, 94°C for 2 minutes; step 2, 94°C for 15 seconds; step 3, 64°C for 12 minutes; step 4, go back to step two 20 times; step 5, 72°C for 10 minutes; step 6, melting curve from 65°C to 95°C; step 7, hold at 4°C indefinitely.

mtDNA deletion assay

The amplification of mtDNA deletion products was performed as previously described [7]. Primers used to amplify the deletion junction for two common mitochondrial deletions are listed in Supplementary Table 2. The thermal cycling protocol used was 95°C for 20 seconds, 55°C for 45 seconds, 72°C for 90 seconds for 35 cycles. A 3′-5′ DNA proofreading-deficient MEF cell line generated by a p.D181A point mutation in the mtDNA polymerase (PolG) was used as a control for the generation of mtDNA deletions under H_2O_2 exposure. This PolG MEF cell line was a gift from Dr. David Chan at the California Institute of Technology.

Supplementary Bibliography

1 Rieder, S. E. & Emr, S. D. Overview of subcellular fractionation procedures for the yeast Saccharomyces cerevisiae. *Curr Protoc Cell Biol* **Chapter 3**, Unit 3 7, doi:10.1002/0471143030.cb0307s07 (2001).

2 Ashley, N., Harris, D. & Poulton, J. Detection of mitochondrial DNA depletion in living human cells using PicoGreen staining. *Exp Cell Res* **303**, 432-446, doi:10.1016/j.yexcr.2004.10.013 (2005).

3 Rando, T. A. & Blau, H. M. Primary mouse myoblast purification, characterization, and transplantation for cell-mediated gene therapy. *J Cell Biol* **125**, 1275-1287 (1994).

4 Nicholls, D. G. *et al.* Bioenergetic profile experiment using C2C12 myoblast cells. *J Vis Exp*, doi:10.3791/2511 (2010).

5 Vermulst, M., Bielas, J. H. & Loeb, L. A. Quantification of random mutations in the mitochondrial genome. *Methods* **46**, 263-268, doi:10.1016/j.ymeth.2008.10.008 (2008).

6 Santos, J. H., Mandavilli, B. S. & Van Houten, B. Measuring oxidative mtDNA damage and repair using quantitative PCR. *Methods Mol Biol* **197**, 159-176, doi:10.1385/1-59259-284-8:159 (2002).

7 Tanhauser, S. M. & Laipis, P. J. Multiple deletions are detectable in mitochondrial DNA of aging mice. *J Biol Chem* **270**, 24769-24775 (1995).

Supplementary Tables and Figures

Supplementary Table 1. Primary antibodies

Name	Application(s)	Source
Anti-human		
TDP1	IB, IF	Abcam (ab4166)
TIM23	IB, IF	Santa Cruz (sc-13297)
TOM20	IB	Abcam (ab56783)
COMPLEX IV	IB	Abcam (ab14744)
HISTONE 2B	IB	Abcam (ab1790)
HISTONE H4	IB	Cell Signaling Technology (#2592)
UBIQUITIN	IB	Cell Signaling Technology (#3933)
GAPDH	IB	Abcam (ab9485)
HA	IB	Thermo Fisher Scientific (#26183)
DNA LIGASE III	IB	Abcam (ab587)
XRCC1	IB	Abcam (ab1838)
ERK1	IB, IF	Cell Signaling Technology (#4372)
P38	IB, IF	Cell Signaling Technology (#9212)
Phosphorylated ERK1	IB	Cell Signaling Technology (#9101)
Phosphorylated P38	IB	Cell Signaling Technology (#9211)
Phosphorylated JNK1/2	IB	Thermo Fisher Scientific (#44-682G)
Anti-mouse		
TDP1	IB, IF	Abcam (ab4166)
TOM20	IB	Santa Cruz (sc-17764)
HISTONE H4	IB	Cell Signaling Technology (#2592)
DNA LIGASE III	IB	Abcam (ab125434)
HA	IB	Thermo Fisher Scientific (#26183)
XRCC1	IB	Abcam (ab1947)
COMPLEX IV	IB	Abcam (ab14744)
Anti-yeast		
GFP	IB	Abcam (ab290)
NSP1P	IB	Abcam (ab4641)
PORIN	IB	Thermo Fisher Scientific (#459500)

Abbreviations: IB, immunoblot; IF, immunofluorescence

Supplementary Table 2. Oligonucleotide sequences.

Name	Sequence (5′ to 3′)
Quantitative PCR primers	
TDP1 - forward	AGGCTAAGGCTCACCTCCAT
TDP1 - reverse	TTCCTGGAGTCTTGCTTTCC
GAPDH - forward	TTAGCACCCCTGGCCAAGG
GAPDH - reverse	CTTACTCCTTGGAGGCCATG
mtCOXII - forward	GATAACCGAGTCGTTCTGCAA
mtCOXII - reverse	CCTGGTCGGTTTGATGTTACT
S6 ribosomal protein - forward	GTCCGCCAGTATGTTGTCAGGAAG
S6 ribosomal protein - reverse	GCTTTGGTCCTGGGCTTCTTACC
Mutation assay primers	
control - forward	TCGGCGTAAAACGTGTCAAC
control - reverse	CCGCCAAGTCCTTTGAGTTT
Taq634 - forward	ACTCAAAGGACTTGGCGGTA
Taq634 - reverse	AGCCCATTTCTTCCCATTTC
nDNA - forward	ATGGAAAGCCTGCCATCATG
nDNA - reverse	TCCTTGTTGTTCAGCATCAC
mtDNA - forward	CCTATCACCCTTGCCATCAT
mtDNA - reverse	GAGGCTGTTGCTTGTGTGAC
mtDNA lesion assay primers	
long fragment - forward	GCCAGCCTGACCCATAGCCATAATAT
long fragment - reverse	GAGAGATTTTATGGGTGTAATGCGG
short fragment - forward	CCCAGCTACTACCATCATTCAAGT
short fragment - reverse	GATGGTTTGGGAGATTGGTTGATG
mtDNA deletion assay primers	
deletion (D1) - forward	CATTCTAGCCTCGTACCAACAC
deletion (D1) - reverse	GATTCGTATGCTGTACATAGCTG
deletion (D13) - forward	AATTACAGGCTTCCGACACA
deletion (D13) - reverse	TGATGTTGGAGTTATGTTGG
Site-directed mutagenesis primers	
S81A - forward	GGCAGAAAAGCGGTGCCCAGGAGGACCTCGGC
S81A - reverse	GGCGAGGTCCTCCTGGGCACCGCTTTTCTGCC

Supplementary Figure Legends:

Supplementary Figure 1: Effect of H_2O_2 treatment on cultured cells. (a) Graph showing the effect of H_2O_2 treatment on the survival of cultured human dermal fibroblasts and mouse embryonic fibroblasts. Human and mouse cells were treated for 1 hour with varying H_2O_2 concentrations before measuring the level of viable cells using the MTT assay. (b) Representative immunofluorescent images showing colocalization of Tdp1 and the mitochondrial dye MitoTracker® in human dermal fibroblasts and mouse embryonic fibroblasts (MEFs) following treatment with 1 μM H_2O_2 for 1 hour. (c) Activation of MAP kinases in human dermal fibroblasts by H_2O_2 treatment. Left panel: Graph showing the level of JNK, ERK1 and P38 phosphorylation following culture with 0, 1 or 2 μM H_2O_2 for 1 hour. Right panel: Picture of an immunoblot of lysates from whole-cell extracts of H_2O_2-treated human fibroblasts probed for p-JNK, p-ERK, and p-P38. ***, $P<0.001$. (d) Graphs showing the expression of P38 (left) and ERK1 (right) by quantitative PCR after treatment with non-targeting siRNA or siERK1/siP38 for 72 hours. Gene expression was normalized to *GAPDH*. Error bars represent one standard deviation for three independent experiments. Scale bar = 10 μm.

Supplementary Figure 2: P38 and ERK1 modulate Tdp1 exit from the nucleus. (a) Dependence of Tdp1 clearance from the nucleus on P38 following H_2O_2 treatment of cultured human fibroblasts. Left panel: Picture of a representative immunoblot of lysates from nuclear extracts of cultured human fibroblasts without or with knockdown of P38 and in the absence or presence of H_2O_2 treatment. Histone H4 was used as a loading control. Right panel: Graph showing fold change in nuclear Tdp1 levels across three independent experiments following treatment with 1 μM H_2O_2 for 1 hour and in the absence or presence of P38 knockdown. Tdp1

levels were normalized to Histone H4 levels and fold changes are shown relative to levels in cells treated with non-targeting siRNA. Error bars represent one standard deviation. (b) Dependence of Tdp1 clearance from the nucleus on ERK1 following H_2O_2 treatment of cultured human fibroblasts. Left panel: Picture of a representative immunoblot of lysates from nuclear extracts of cultured human fibroblasts without or with knockdown of ERK1 and in the absence or presence of H_2O_2 treatment. Histone H4 was used as a loading control. Right panel: Graph showing fold change in nuclear Tdp1 levels across three independent experiments following treatment with 1 μM H_2O_2 for 1 hour and in the absence or presence of ERK1 knockdown. Tdp1 levels were normalized to Histone H4 levels and fold changes are shown relative to levels in cells treated with non-targeting siRNA. Error bars represent one standard deviation. (c) Dependence of Tdp1 clearance from the nucleus on P38 following rotenone treatment of cultured human fibroblasts. Left panel: Picture of a representative immunoblot of lysates from nuclear extracts of cultured human fibroblasts without or with knockdown of P38 and in the absence or presence of rotenone treatment. Histone H4 was used as a loading control. Right panel: Graph showing fold change in nuclear Tdp1 levels across three independent experiments following treatment with 200 nM rotenone for 1 hour and in the absence or presence of P38 knockdown. Tdp1 levels were normalized to Histone H4 levels and then fold changes are shown relative to levels in cells treated with non-targeting siRNA. Error bars represent one standard deviation. (d) Dependence of Tdp1 clearance from the nucleus on ERK1 following rotenone treatment of cultured human fibroblasts. Left panel: Picture of a representative immunoblot of lysates from nuclear extracts of cultured human fibroblasts without or with knockdown of ERK1 and in the absence or presence of rotenone treatment. Histone H4 was used as a loading control. Right panel: Graph showing fold change in nuclear Tdp1 levels across three independent experiments

following treatment with 200 nM rotenone for 1 hour and in the absence or presence of ERK1 knockdown. Tdp1 levels were normalized to Histone H4 levels and then fold changes are shown relative to levels in cells treated with non-targeting siRNA. (e) Graph showing detection of random Taq1 mutation sites in the mtDNA of human dermal fibroblasts with or without treatment with siRNA and 1 μM H_2O_2 for 96 hours. Error bars represent one standard deviation. **, $P<0.01$; ***, $P<0.001$.

Supplementary Figure 1

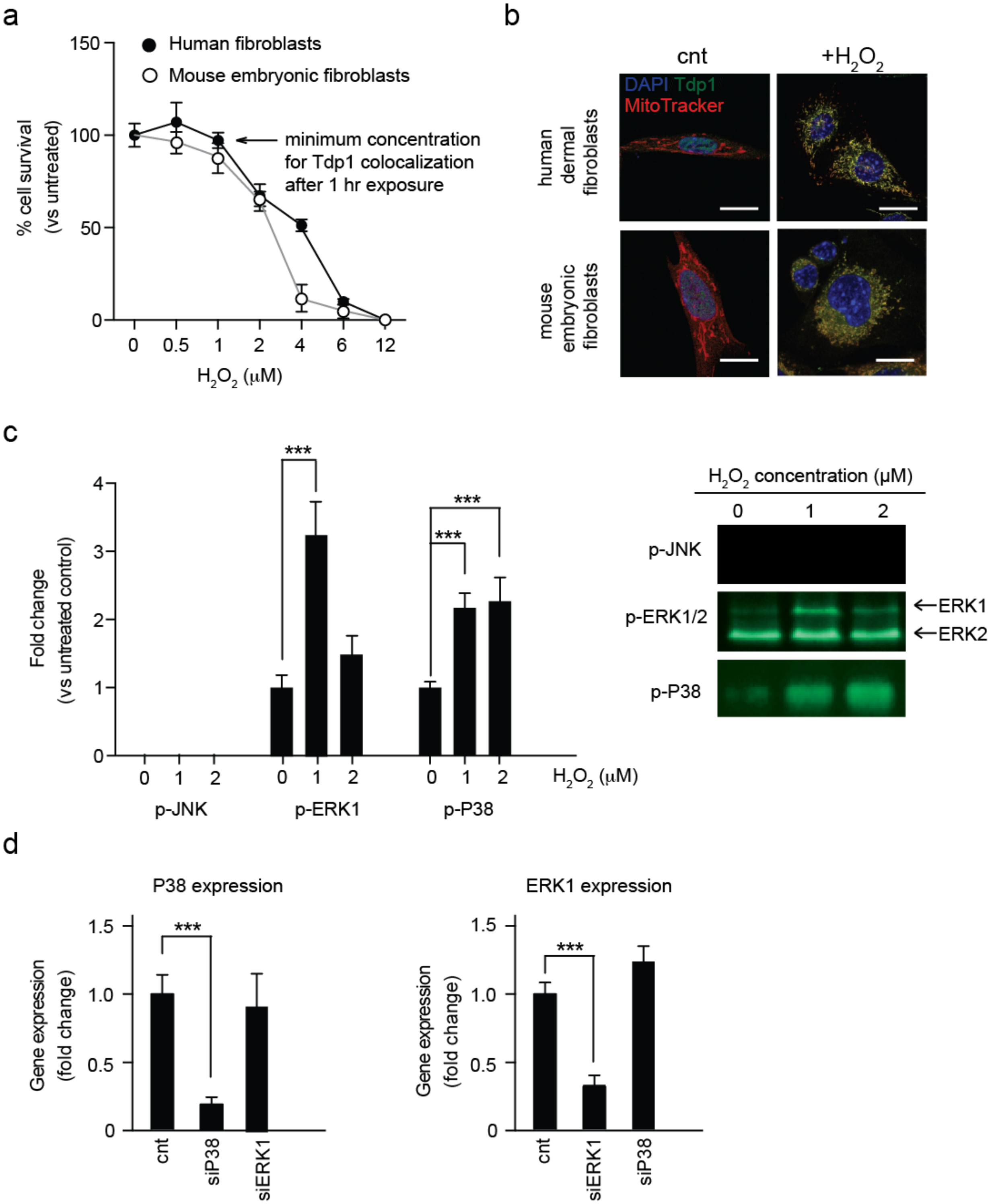

Supplementary Figure 2

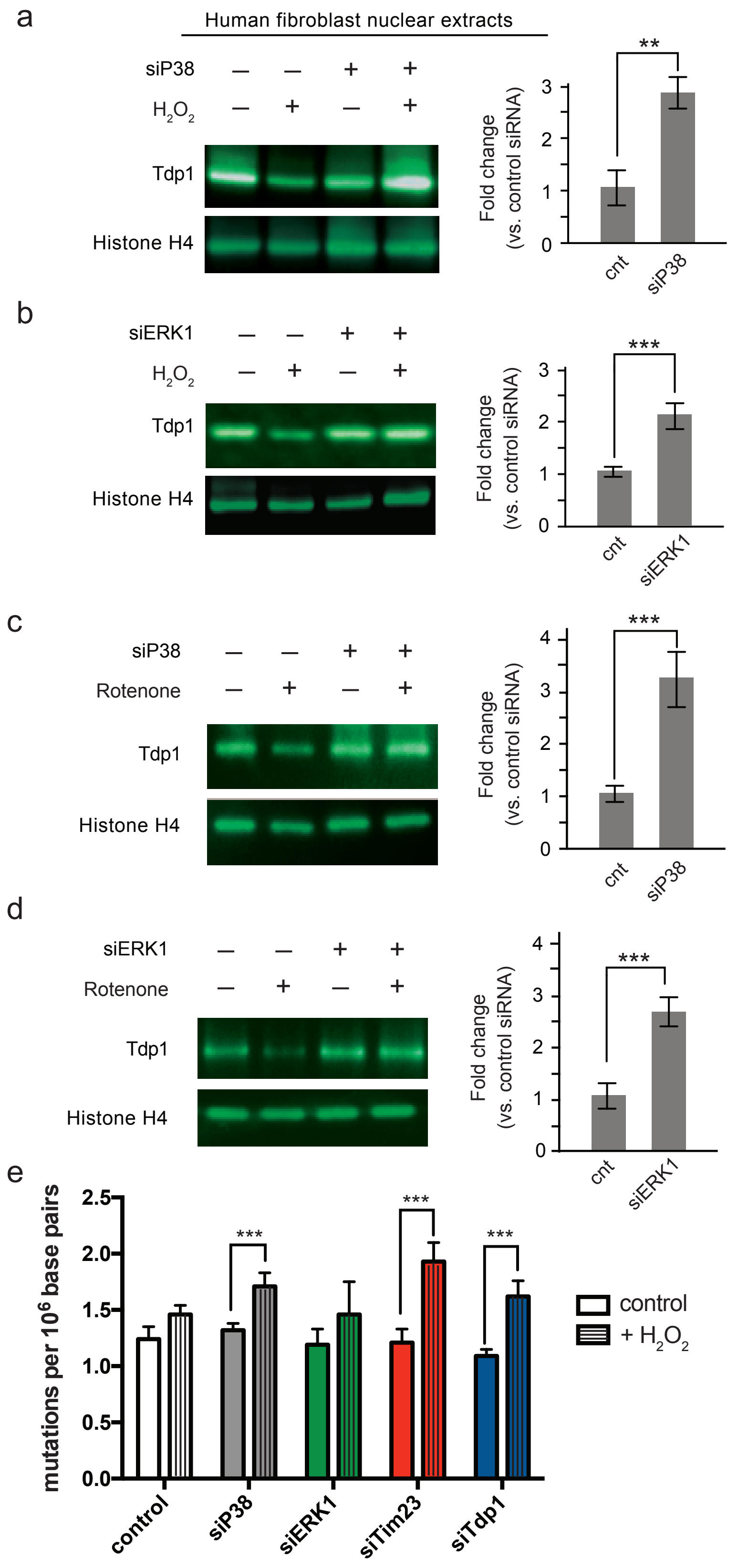

Supplement: Supplementary file 1 — Supplementary Material [file 41598_2018_22547_MOESM1_ESM.pdf]
